# A local, non-commercial tissue bank connected to an organ donor program can produce musculoskeletal allografts of uniform quality at very low costs – ten years’ experience

**DOI:** 10.1007/s10561-024-10151-2

**Published:** 2024-11-23

**Authors:** Helia Azkia, Lene H. Harritshøj, Connie Nielsen, Niels Agerlin, Mette G. Jensen, Jens G. Hillingsø, Pia C. Andersen, Michael R. Krogsgaard

**Affiliations:** 1 Section for Sports Traumatology, Department of Orthopedic Surgery, Copenhagen University Hospital Bispebjerg, Hospital Bispebjerg, 2400 Copenhagen NV, Denmark; 2 Department of Clinical Immunology, Copenhagen University Hospital, Rigshospitalet, Copenhagen, Denmark; 3 Department of Neurosurgery, Copenhagen University Hospital Rigshospitalet, Copenhagen, Denmark; 4 Transplantation Coordinator, Copenhagen University Hospital Rigshospitalet, Copenhagen, Denmark; 5 Department of Clinical Medicine, Faculty of Health and Clinical Sciences, Copenhagen University, Copenhagen, Denmark

**Keywords:** Allograft, Musculoskeletal, Bank, Organ donors, Bacterial contamination

## Abstract

It is common practice that allograft tissues for knee multiligament reconstruction, meniscus transplantation, cartilage replacement and other advanced procedures are made available through commercial banks. The aim was to present the 10-year experience with a local, non-commercial tissue bank, established in 2014. The allograft bank was connected to an existing organ donor program. Following organ procurement, the musculoskeletal tissue is removed and stored immediately, fresh frozen to − 80 degrees Celsius (except hyaline cartilage, which is stored at 5 degrees Celsius). The donor is tested for contagious disease and the grafts for bacteria. When all results are negative, the grafts are released. When thawed before use a swap is cultured. Consecutive, prospectively collected data were analyzed. There were 31 donations, resulting in 1160 grafts. Only 40 grafts (3.4%) had a positive bacteria culture and were discarded. 552 recipients have been treated by use of these allografts. All grafts had negative bacterial cultures in swaps obtained before thawing, and there were no recorded transplantation related complications. The expenses for local grafts were 10–15% of the costs for grafts obtained from foreign banks. Through the local donation program, it was possible to establish a tissue bank with controlled quality grafts at minimal costs, minimizing the need for transportation of frozen grafts retrieved in other countries. Centers for specialized orthopedic surgery using allograft tissue can be self-providing and reduce costs by retrieving and handling allograft tissues locally.

**Supplementary Information:**

The online version contains supplementary material available at 10.1007/s10561-024-10151-2.

## Introduction

**Fig. 1:**
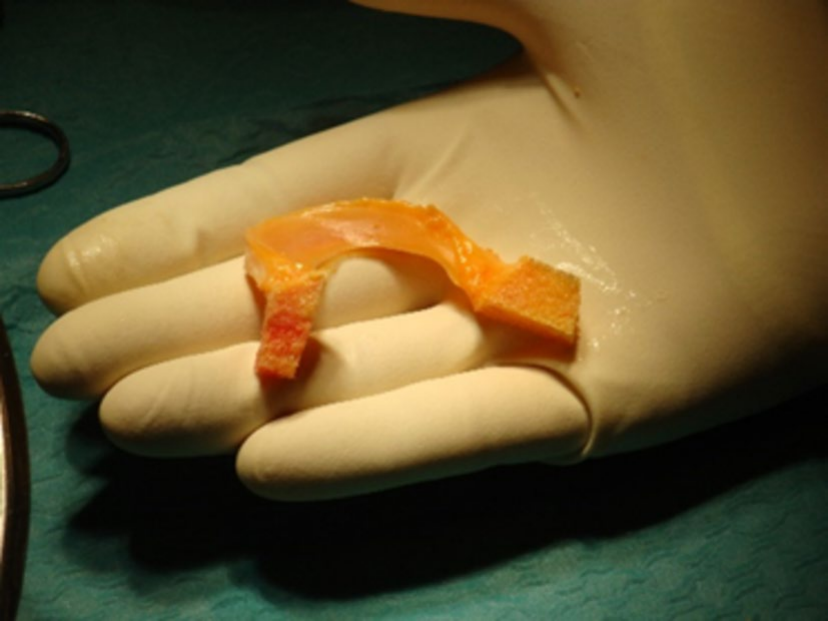
Allograft medial meniscus

**Fig. 2:**
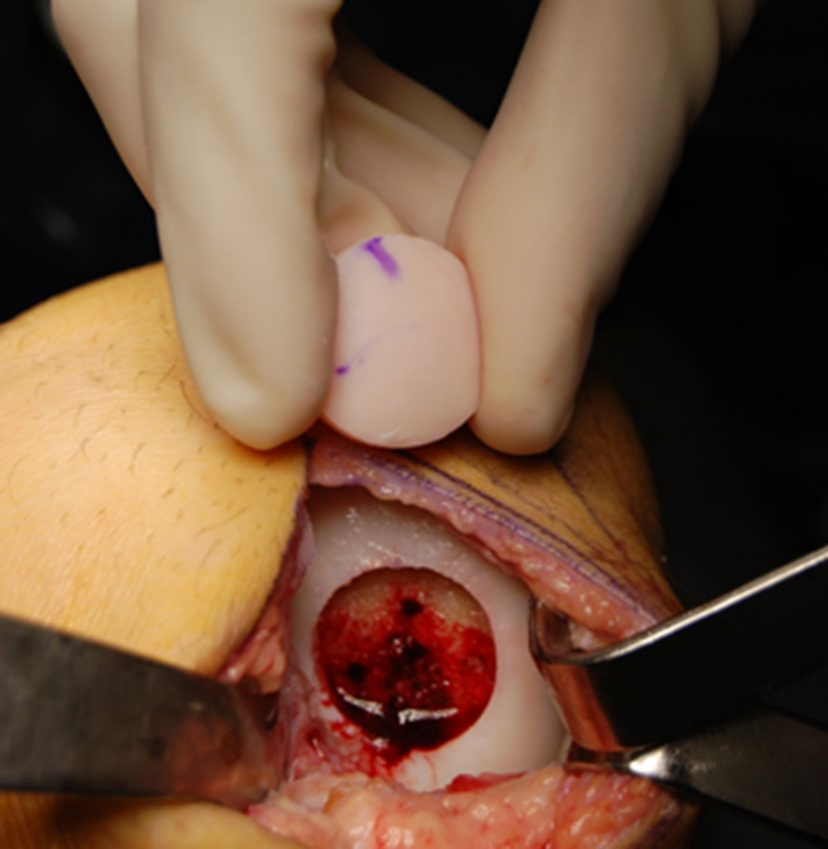
Fresh allograft cartilage transplantation (mega-OATS) to the medial femoral condyle

**Fig. 3:**
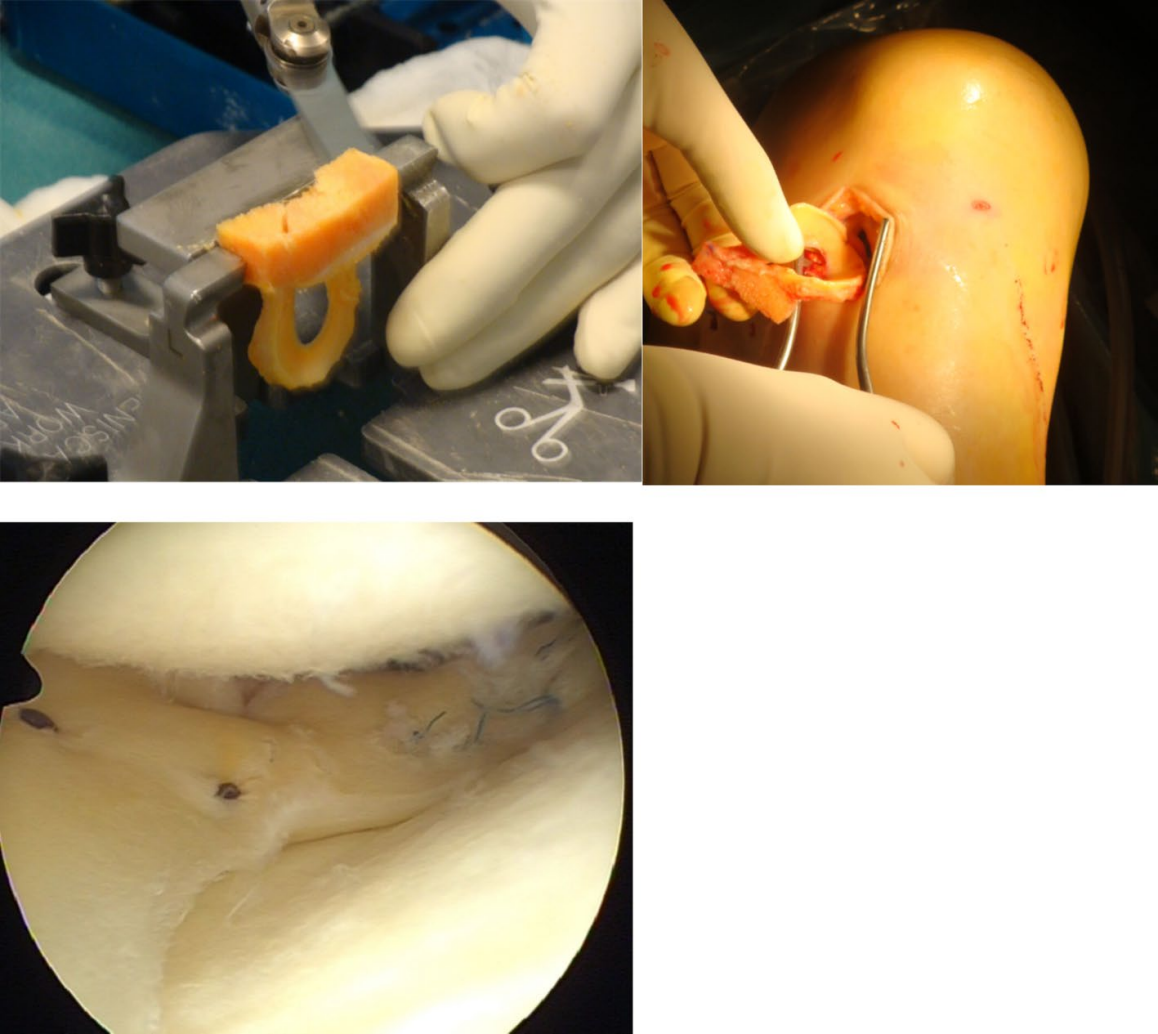
Lateral meniscus transplantation, using an allograft. First photo: shaping the bone block of the allograft. Second picture: introducing the graft into the knee. Third photo: arthroscopic view of the implanted meniscus allograft

Graft tissue is necessary for optimal treatment of several musculoskeletal conditions, in which repair is not possible or tissue is not able to regenerate. Grafts from the patient (autografts) are preferred except when removal of autograft tissue would result in disabling donor site problems, or when autograft tissue is not available (Figs. 1, 2 and 3). In such cases tissues from human donors (allografts) are useful.

Allografts have been used for decades with good results (Erivan et al. 2018) and are commercially available in most countries. The clinical outcomes of surgery are independent of whether autografts or allografts have been used (Dhillon et al. 2022).

In the US allografts are often sterilized chemically or by irradiation, whereas in Europe they are decontaminated in antibiotic solutions or used fresh frozen. High doses of irradiation weaken the collagen tissue in allografts (Lansdown et al. 2017), and chemical decontamination may change strength and mechanical properties of the tissue (Lansdown et al. 2017). Donor age > 40 years has a small negative effect on the mechanical properties of tendon grafts (Swank et al. 2015).

With the progressive use of advanced surgical procedures, the need for allogenic connective tissue (grafts) has been increasing. It is possible to obtain allografts from tissue banks in other countries, but from an ethical point of view Denmark should best be self-providing and it can be argued that human tissue should not be a commercial product. Also, differences in how donors are selected, and grafts are obtained and preserved might affect the tissue quality and the risk of contamination (Vehmeyer et al. 2002).

Therefore, work on a local, non-commercial tissue bank was initiated in 2012, and the bank was established in June 2014. The purpose was to make grafts of the highest possible quality available for patient treatment at the lowest possible cost.

The aim of this study was to report the 10-year experience of this tissue bank, based on the prospectively collected data in the bank registry.

## Materials and methods

The establishment of the tissue bank was a collaboration between Section for Sports Traumatology at Copenhagen University Hospital Copenhagen–at which the surgical treatment of highly specialized sports traumatology is centered for Eastern Denmark–and Department of Clinical Immunology, Department of Neurosurgery and the Transplantation Coordinator, Copenhagen University Hospital Rigshospitalet, Denmark.

Removal of musculoskeletal allograft tissue does not require that the donor is alive. Removal within 24 h after death is usually regarded safe in relation to degeneration and contamination of the tissue. Therefore, it was discussed to obtain grafts from severely injured patients who were dead at admission or died shortly after admission to the level 1 trauma center at the National Hospital in Copenhagen. This would make it possible to have a relatively long notice in relation to remove tissue, but these patients are not routinely screened for infectious diseases, the patient history might be more difficult to obtain, and the acute, dramatic circumstances might make a request to the relatives regarding donation of tissue inappropriate in many cases. It was more logical to connect removal of allografts to the organ donor program in Copenhagen. This collaboration was established with the Department of Neurosurgery, the Organ Transplantation Coordinating Center, and The Tissue Establishment (TE) at the Department of Clinical Immunology. The allograft bank permission was obtained from the Danish Medical Agency, who administered the EU directive derived Danish tissue transplantation law (described in the supplementary material). The organ donation consent form was extended to include an option to donate musculoskeletal tissue. It is the responsibility of the head of the orthopedic team to go through the medical files of the donor to make sure that the donor is suitable for tissue donation (described in the supplementary material), and to document this in a checklist of donor criteria. The procedure – which is still functional - is that after the permission from relatives has been obtained, and the obligate test results of contagious infections are negative, the transplantation coordinator contacts the orthopedic team (two surgeons and two surgical nurses) and following organ procurement and suture of all incisions from this, the donor is extubated, and the musculoskeletal tissue is removed. Only the allograft team of two orthopedic surgeons and two surgical nurses are allowed in the operating theatre during the allograft donation. The operative procedure is described in the supplementary material. The age limit for tissue donors was set to 50 years for tendons, 40 years for menisci and 30 years for hyaline cartilage. Routinely, grafts are only removed from the legs (supplementary material), but for special purposes tissue can be removed from the arms. The donor is tested for infectious diseases according to the standards in the Scandiatransplant project, described in detail in the supplementary material. Material for microbiological culturing is obtained by a swap from each allograft, which is then placed in sterile double plastic jars. The tissue is handled, stored and registered by the TE at the Department of Clinical Immunology immediately after the donation, and fresh frozen to − 80 degrees Celsius (except hyaline cartilage, which is stored at 5 degrees Celsius in a nutrient medium). The donor has been tested for obligate contagious disease and released with negative results by the TE. The swap is cultured for 5 days. Following negative results of the microbiological culturing the grafts are released for use. Grafts with a positive culture are discarded.

Full relevant information about the donor is registered at the TE, where all grafts and recipients are registered in the Laboratory Information System to achieve traceability and biovigilance between donor and recipients, which is obligate for a 30-year period. An anonymous list of released grafts with information of size, date of procurement and Rhesus status of the donor is made available for the Section of Sports Traumatology at Copenhagen University Hospital Bispebjerg for selection of the best suited allografts for specific patients/recipients. The graft number is also registered in the medical file of the recipient along with the indication (e.g., reconstruction of the posterior cruciate ligament). Grafts are cultured when they are used for transplantation by swap sampling immediately after removal from the sterile container and before they are thawed in a 20 degrees Celsius solution with 360 mg Gentamycin/liter.

This study was performed according to STROBE checklist for cohort studies. Data were retrieved from the registries at TE and Section for Sports Traumatology. Each graft has a unique number to which information on date of donation, graft type and microbiology at removal is registered by TE, and information on the recipient, including microbiology at the operation and what the graft was used for is registered at Section for Sports Traumatology. These data are presented descriptively.

In cases when a necessary specific allograft is not available from the local bank, it is bought from one of two TEs in the EU.

The expenses for the orthopedic team, plastic jars, culturing, storage, and handling are pooled for each donor and an average price per released graft to cover these expenses is calculated. When a graft is used for the treatment of a recipient these costs are reimbursed. The average cost for a graft is about 150 EUR. The cost of a graft from a TE outside the country varies from 800 to 1,300 EUR, depending on the type of graft, and transportation is about 320 EUR.

This research received no specific grant from any funding. agency in the public, commercial, or not-for-profit sectors. All authors declare no conflict of interest.

Ethical permission was not required for this study, but permission to register data was obtained.

Large Language Models and Artificial Intelligence have not been used in connection with the study and writing of the manuscript.

## Results

The first donation was achieved on 12 July 2014 and there has been a total of 31 donations, resulting in 1160 grafts. Forty grafts (3,4%) were discarded due to a positive bacteria culture (Table 1). 552 recipients have been treated by use of the allografts: One hundred and seventy five knee multi-ligament reconstructions, 226 revision ligament reconstructions, 44 meniscal transplantations, 18 fresh cartilage transplantations, 3 tibial plateau + meniscal transplantations, 9 sternoclavicular- and 1 patellofemoral joint stabilisation, 4 quadriceps reconstructions, 9 labral reconstructions (hip), 2 pectoralis major tendon reconstructions, 1 revision ankle stabilisation in a patient with Ehlers-Danlos syndrome, and 45 one ligament reconstructions (knee). During this 10-year period it was necessary in addition to buy 245 grafts from TEs abroad because local supply was not sufficient.

**Table 1:** Grafts with a positive microbiological culture immediately after removal from donor. The number of present organisms were graded as 4 + (many, heavy growth), 3 + (moderate growth), 2 + (few or light growth) and 1 + (rare)

Donor number	Number of grafts with positive culture in each donor	Graft	Microbiological findings and grading of growth
0	1	Tibialis posterior tendon, left	Staphylococcus Epidermidis +
1	3	Extensor hallucis longus, left	Moxarelle catarrharlis + Staphylococcus Hominis +
1	3	Medial collateral ligament, left	Coagulase negative Stahylococcus +
1	3	Medial meniscus, left	Coagulase negative Stahylococcus +
2	1	Iliotibial tendon, right	Micrococcus luteus +
3	0		
4	0		
5	7	Iliotibial tendon, part 1/3, left	Micrococcus luteus + Acineto lwoffii
5	7	Biceps femoris tendon, left	Staphylococcus epidermidis +
5	7	Lateral meniscus, left	Staphylococcus epidermidis +
5	7	Flexor digitorum longus tendon, left	Staphylococcus epidermidis +
5	7	Biceps femoris tendon, right	Staphylococcus epidermidis +
5	7	Lateral meniscus, right	Staphylococcus epidermidis +
5	7	Achilles tendon, right	Staphylococcus epidermidis +
6	0		
7	1	Medial collateral ligament, left	Staphylococcus capitis +
8	0		
9	0		
10	2	Peroneus longus tendon, left	Staphylococcus epidermidis +
10	2	Quadriceps tendon, left	Staphylococcus epidermidis +
11	0		
12	0		
13	0		
14	1	Tibialis anterior tendon, right	Staphylococcus epidermidis +
15	1	Medial meniscus, left	Staphylococcs epidermidis +
16	1	Patellar tendon, half, right	Normal skin flora
17	4	Quadriceps tendon, left	Haemofilus parainfluenzae +
17	4	Flexor digitorum longus, left	Staphylococcis hominis +
17	4	Patellar tendon, half, right	Staphylococcus capitis +
17	4	Achilles tendon, right	proprionibacterium acnes +
18	0		
19	2	Quadriceps tendon, left	Staphylococcus capitis +
19	2	Biceps femoris tendon, left	Staphylococcus capitis + +
20	5	Gracilis tendon, left	Staphylococcus epidermidis +
20	5	Tibialis posterior tendon, left	Brevibacterium species (Brevibacterium ravenspurgense)
20	5	Peroneus brevis tendon, right	corynebacterium afermentas
20	5	Extensor tendon, undefined, right	Staphylococcus capitis +
20	5	Tibialis posterior tendon, right	Staphylococcus capitis +
21	0		
22	0		
23	1	Iliotibial tendon, left	Staphylococcis warneri
24	0		
25	4	Semitendinosus tendon, left	Staphylococcus capitis +
25	4	Flexor hallucis longus tendon, left	Micrococcus luteus +
25	4	Achilles tendon, left	Staphylococcus capitis +
25	4	Tibialis posterior tendon, right	Chryseobacterium species +
26	1	Flexor hallucis longus tendon, left	Staphylococcus hominis +
27	0		
28	1	Gracilis tendon, right	Bacillus pumilus
29	2	Extensor digitorum communis, right	Staphylococcus hominis +
29	2	Achilles tendon, right	Cutibacterium acnes
30	3	Quadriceps tendon, left	Staphylococcus hominis +
30	3	Extensor digitorum longum, right	Staphylococcus epidermidis +
30	3	Peroneus longus tendon, right	Micrococcus luteus
31	0		
32	1	Semitendinosus tendon, right	Micrococcus luteus

All grafts had negative bacterial cultures in swaps obtained right before thawing, and there were no recorded transplantation related complications.

## Discussion

This study shows that it is possible locally in relation to a solid-organ transplantation program to establish a tissue allograft bank of a dimension which is relevant to support a center for highly specialized sports traumatological surgery, like knee multiligament reconstruction, meniscal replacement and other procedures that cannot be performed without access to allograft tissue. Ethically, it is optimal that regions are self-providing in relation to allograft tissue, and it is possible locally to define standards for donors, retrieval of tissue and handling of tissue on top of requirements from national and international regulations. In addition, it is a cost-efficient initiative, and it reduces the need for transportation of frozen grafts over long distances. An allograft bank has been established after the same principles at the other highly specialized center for sports traumatology in Denmark (at Skejby University Hospital in Aarhus, information from professor Martin Lind).

In the current allograft bank set-up, the tissue was removed shortly after termination of cardiac activity in organ donors, and it was fresh frozen within few hours after removal. The surgical team of four were the only persons present in the operation room. This is optimal in relation to the risk for contamination of the allograft tissue, as hours from removal of the heart (cadaver time), non-organ donors (meaning persons in whom donation started after cardiac arrest) and number of persons involved in retrieval of tissue all increase this risk (Vehmeyer et al. 2002 and Paolin et al. 2017a). There are reports of very high rates of contamination in tissue allografts-as high as 35% in organ donors and 55% in non-organ donors (Paolin et al. (2017b)), but in most reported cohorts it is much lower: 12.6% in bone allografts (Baseri et al. 2022) and 10–21% in tendon allografts (Schubert et al. 2012 and Viñuela-Prieto et al. 2019), though much higher than our finding of 3.4%. The contamination rates are affected by sampling technique, as swap samples are twice as often cultured positive compared to tissue samples (Baseri et al. 2022). Most of the relevant bacteria are cultured within 5 days, but Proprionebacteria are usually only detected after prolonged culturing (Drago et al. 2015) and may not have been detected in the current setting. Also, the time from the graft is exposed in the donor and until it is placed in the sterile container is influencing the risk of contamination.

In literature, bacteria with high pathogenicity have been reported in 3% of grafts, and this rate could not be reduced by rinsing the graft in an antibacterial solution (Deijkers et al. 1997).

The bacteria that were cultured were typically skin flora. Donor 5 had 7 positive cultures (Table 1) which was an unacceptably high number. We found no specific reason for this and after having discussed the surgical procedure we found no reason to change practice.

The rate of contamination is higher in other tissues (skin, heart valves, vascular grafts) compared to musculoskeletal grafts (Paolin et al. 2017a, Georges et al. 2021, Louart et al. 2019). Even though reported cases of infection and other adverse effects to allograft implantation are extremely sparse and mainly related to allograft banking in the last century under conditions that are not comparable to now-a-days regulations (Hinsenkamp etal. 2012) several cases of Clostridium Perfringens in patients who had received allografts from one specific graft bank were reported around the turn of the century (Kainer et al., 2004), underlining the importance of sufficient retrieval and handling of grafts. However, in series of patients receiving fresh frozen bone allograft tissue the postoperative infection rate was 2.6%, which was not regarded as higher than in comparable patients who had not received allograft tissue (Man et al. 2016). In another series, 5–10% of tendon allografts had a positive peroperative bacterial culture when the graft was used, but none of the recipients developed infection, despite no additional antibiotic treatment (Guelich et al. 2007, Phornphutkul et al. 2009, Schmidt-Hebbel et al. 2018). Therefore, with retrieval and handling of allografts after now-a-days principles, the risk for disease transmission seems negligible.

However, Staphylococcus and many other bacteria and vira can survive − 80 degrees Celsius freezing (Panisello Yagüe et al. 2021). Therefore, it is a common procedure to decontaminate the allografts through antibiotic soaking, irradiation, or chemical additives. According to some algorithms the grafts are kept at 4 degrees Celsius for several days while they are soaked in antibiotic solutions before they are frozen to -80 degrees Celsius (Paolin et al. 2017b) to optimise the effect of antibiotics, but despite this, there is still a significant proportion of allografts with a positive culture (Paolin et al. 2017a). Collagen tissue loses some of its mechanical properties following gamma-irradiation, in particular at doses > 2.5 Mrad (DiBartola etal. 2016). However, the future might show that radiation by electrons have a smaller negative influence on collagens and could be used to sterilize allografts (Hoburg et al. 2010). In the current allograft bank, it was decided not to use any decontamination, except thawing of the allografts peroperatively in a Gentamycin solution. Only 3.4% of the grafts were discarded due to a positive culture right after retrieval, and this is a small proportion compared to published experience, showing that the algorithm of the current allograft bank is satisfactory.

## Conclusion

Through the established local donation program, it has been possible to establish a tissue bank with high quality grafts at minimal costs, minimizing the need for transportation of frozen grafts, retrieved in other countries. The number of rejected grafts was minimal and there were no identified transplantation related complications in 552 recipients treated with more than 1,000 allografts.

Centers for highly specialized orthopedic surgery, dependent on allograft tissue from banks abroad for operative treatments, can be self-providing and reduce costs by retrieving and handling allograft tissue locally in collaboration with an organ-donor program.

## Supplementary Information

Below is the link to the electronic supplementary material.

Supplementary file1 (DOCX 15 kb)

Supplementary file2 (DOCX 14 kb)

Supplementary file3 (DOCX 19 kb)

## Data Availability

No datasets were generated or analysed during the current study.
