# Supplementary material for: A local, non-commercial tissue bank connected to an organ donor program can produce musculoskeletal allografts of uniform quality at very low costs – ten years’ experience
Source: Cell Tissue Bank. 2024 Nov 23;26(1):1. doi: 10.1007/s10561-024-10151-2 (PMC11584506; doi:10.1007/s10561-024-10151-2)
Supplement: Supplementary file 1 — Supplementary file1 (DOCX 15 kb) [file 10561_2024_10151_MOESM1_ESM.docx]

Criteria for exclusion of a donor.

The patient cannot be used for allograft donation if any of these conditions are present:

1. Malignant disease – earlier or present, except basalcell carcinoma of the skin and carcinoma in situ on cervix.

2. Wounds or other kinds of contamination of the donation field (in most cases the legs).

3. Conditions that can weaken the grafts: Earlier operation in both knees (if only in one knee the other can be used for donation). Rheumatoid arthritis or any other systemic autoimmune condition.

4. Systemic infection or local, ongoing infection of tissue in the donation field.

5. Anamnestic information about earlier or present hepatitis B- hepatitis C- or HTLV-1/2-infection.

6. Anamnestic information about HIV infection.

7. Any risk behavior related to HIV, hepatitis B or hepatitis C: intravenous drug addiction, sex between men, prostitution, donor has received blood or tissue in a foreign country during the past 6 months, donor has got a tattoo within the last 6 months.

8. Any risk behaviour for Creutz-Jacobs disease/variant CJD: Treated with growth hormone before 1994, donor has received transplantation if dura mater or cornea, unexplained rapidly progressing dementia or unexplained neurological conditions, Creutzfeld-Jacobs disease in family members, donor has lived in the United Kingdom for more than a year between 1980 and 1996.

9. Unexplained fever in relation to travel outside Europe during the past 6 months.

10. Travel outside the Nordic countries, UK or Germany within the past month before donation (risk for insect-transmitted infections) .

11. Suspicion of any serious disease of unknown nature.

12. Risk for xenogene disease transmission (donor has received living tissue from an animal).

13. Vaccination with live attenuated vaccine within the past 4 weeks (e.g., yellow fever, MFR-vaccine, typhus oral vaccine).

14. Exposition for toxic substances that can accumulate in connective tissue or bone.
