# Supplementary material for: A local, non-commercial tissue bank connected to an organ donor program can produce musculoskeletal allografts of uniform quality at very low costs – ten years’ experience
Source: Cell Tissue Bank. 2024 Nov 23;26(1):1. doi: 10.1007/s10561-024-10151-2 (PMC11584506; doi:10.1007/s10561-024-10151-2)
Supplement: Supplementary file 2 — Supplementary file2 (DOCX 14 kb) [file 10561_2024_10151_MOESM2_ESM.docx]

Screening to exclude disease:

Anti-HIV

HBsAg

anti-HBc

anti-HCV

SARS-CoV-2

anti-CMV IgG

anti-EBV

anti-HSV-1/2

anti-Treponema pallidum

anti-Toxoplasmosis

IGRA

Serum is stored for 10 years to make further analyses possible.

A patient who has traveled or resided in an area with an ongoing outbreak of hemmorrhagic fever during the past two months cannot be a donor.

If the patient has traveled or resided in an area with high prevalence of HTLV-I/II infections (Japan, South America, the Caribbean, the Melanesian islands, Papua New Guinea, the Middle East, or Western, Central and South Africa) an anti-HTLV-I/II is performed and if positive, the patient cannot be donor.

Donors with residency or long term stay in Sub-Saharan Africa during the past five years are screened for malaria by PCR/LAMP. Febrile patients with stay during the past 6 months in any malaria-endemic region are screened for malaria by microscopy. Patients with malaria cannot be allograft donors.

Patients originating from or frequently visiting areas with > 5% prevalence of Strongyloides stercoralis are serologically screened for this and if positive cannot be donors.

Patients originating from or with stay >3 months in countries with risk for transmission of Trypanosoma cruzi (Latin America) are tested for Trypanosoma cruzi IgG. If positive the patient cannot be allograft donor.
