# Supplementary material for: A local, non-commercial tissue bank connected to an organ donor program can produce musculoskeletal allografts of uniform quality at very low costs – ten years’ experience
Source: Cell Tissue Bank. 2024 Nov 23;26(1):1. doi: 10.1007/s10561-024-10151-2 (PMC11584506; doi:10.1007/s10561-024-10151-2)
Supplement: Supplementary file 3 — Supplementary file3 (DOCX 19 kb) [file 10561_2024_10151_MOESM3_ESM.docx]

**Regulations related to allograft transplantation.**

The basis for the Danish regulations related to allograft tissue is the European regulation 2004/23/EF of March 31, 2004 on standards for the quality and safety of donation, collection, testing, processing, preservation, storage and distribution of human tissues and cells. Centers for allograft tissue must be approved by the Danish Health Authority, which comes for physical inspection at intervals, and the activity must be reported to the Danish Health Authority on a yearly basis. Any serious incident must be reported to the Danish Health Authority.

The Danish Law 273 dated 01/04/2006 specifies that tissue centers must be authorized and that persons handling the tissue must have sufficient qualifications.

​Donors must be 15 years or older. All Danes aged 15 and over can indicate in the Danish Organ Donor Register whether they wish to donate their organs and tissue or refuse to do so. For persons between 15 and 18 years of age, their next of kin must also give consent when donating. If a patient and potential donor has not made a decision, it will be the next of kin who makes the decision on whether the patient will be donor and which organs/tissues that can be donated.

In Denmark death is defined by irreparable cessation of all brain function (brain death) or irreversible cessation of breathing and heart activity (circulatory death). Donors are declared dead according to one or both of the criteria, but until March 2023 only brain-dead patients could be donors. Since March 2023 donation is possible in case of death according to any of the two criteria.

The allograft donation is performed in a standard operating theatre at department for abdominal surgery. The donor is prepared for allograft donation like any patient before an operation: the skin of the donor is decontaminated twice by chlorhexidine and the legs are sterilely covered. Only the surgical team is allowed in the operating theatre and they are wearing standard surgical clothing and double surgical glowes, except the person on the floor, handling plastic jars, specimens for culturing etc. All are wearing hats and surgical masks. Allograft tissue is removed from the leg through three incisions: one anterior on the knee, one anterolateral on the lower leg and foot and one posteromedial on the lower leg and foot.

**Criteria for exclusion of an allograft donor.**

The patient cannot be used for allograft donation if any of these conditions are present:

1. Malignant disease – earlier or present, except basal cell carcinoma of the skin and carcinoma in situ on cervix.

2. Wounds or other kinds of contamination of the donation field (in most cases the legs).

3. Conditions that can weaken the grafts: Earlier operation in both knees (if only in one knee the other can be used for donation). Rheumatoid arthritis or any other systemic autoimmune condition.

4. Systemic infection or local, ongoing infection in tissue in the donation field.

5. Anamnestic information about earlier or present hepatitis B- hepatitis C- or HTLV-1/2-infection.

6. Anamnestic information about HIV infection.

7. Any risk behavior related to HIV, hepatitis B or hepatitis C: intravenous drug addiction, sex between men, prostitution, donor has received blood or tissue in a foreign country during the past 6 months, donor has got a tattoo within the last 6 months.

8. Any risk behaviour for Creutz-Jacobs disease/variant CJD: Treated with growth hormone before 1994, donor has received transplantation if dura mater or cornea, unexplained rapidly progressing dementia or unexplained neurological conditions, Creutzfeld-Jacobs disease in family members, donor has lived in the United Kingdom for more than a year between 1980 and 1996.

9. Unexplained fever in relation to travel outside Europe during the past 6 months.

10. Travel outside the Nordic countries, UK or Germany within the past month before donation (risk for insect-transmitted infections).

11. Suspicion of any serious disease of unknown nature.

12. Risk for xenogene disease transmission (donor has received living tissue from an animal).

13. Vaccination with live attenuated vaccine within the past 4 weeks (e.g., yellow fever, MFR-vaccine, typhus oral vaccine).

14. Exposition for toxic substances that can accumulate in connective tissue or bone.

**Screening of potential allograft donors to exclude disease:**

Anti-HIV

HBsAg

anti-HBc

anti-HCV

SARS-CoV-2

anti-CMV IgG

anti-EBV

anti-HSV-1/2

anti-Treponema pallidum

anti-Toxoplasmosis

IGRA

Serum is stored for 10 years to make further analyses possible.

A patient who has traveled or resided in an area with an ongoing outbreak of hemmorrhagic fever during the past two months cannot be an allograft donor.

If the patient has traveled or resided in an area with high prevalence of HTLV-I/II infections (Japan, South America, the Caribbean, the Melanesian islands, Papua New Guinea, the Middle East, or Western, Central and South Africa) an anti-HTLV-I/II is performed and if positive, the patient cannot be an allograft donor.

Donors with residency or long term stay in Sub-Saharan Africa during the past five years are screened for malaria by PCR/LAMP. Febrile patients with stay during the past 6 months in any malaria-endemic region are screened for malaria by microscopy. Patients with malaria cannot be allograft donors.

Patients originating from or frequently visiting areas with > 5% prevalence of Strongyloides stercoralis are serologically screened for this and if positive cannot be allograft donors.

Patients originating from or with stay >3 months in countries with risk for transmission of Trypanosoma cruzi (Latin America) are tested for Trypanosoma cruzi IgG. If positive, the patient cannot be an allograft allograft donor.

**Allograft tissue from each donor:**

Grafts removed from each leg. All donors were < 50 years of age. Meniscal grafts were only removed in donors < 40 years of age and cartilage (femoral condyles) only in donors < 30 years of age.

Quadriceps tendon with bone block

½ patellar tendon with bone blocks in both ends, medial

½ patellar tendon with bone blocks in both ends, lateral

Semitendinosus tendon

Gracilis tendon

Medial collateral ligament with bone blocks in both ends

Medial meniscus with tibial plateau

Lateral meniscus with tibia plateau

Biceps tendon with bone block

Tibialis anterior tendon with bone block

Tibialis posterior tendon with bone block

Achilles tendon with bone block

Flexor hallucis longus tendon

Flexor digitorum longus tendons

Peroneus longus tendon

Peroneus brevis tendon

Iliotibial tendon (occasionally divided into three allografts for reconstruction of the anterior lateral ligament of the knee)

Fresh femoral condyle, lateral

Fresh femoral condyle, medial
